# *Pseudomonas capsici* sp. nov., a plant-pathogenic bacterium isolated from pepper leaf in Georgia, USA

**DOI:** 10.1099/ijsem.0.004971

**Published:** 2021-08-23

**Authors:** Mei Zhao, Santosh Koirala, Hsiao-Chun Chen, Ronald Gitaitis, Brian Kvitko, Bhabesh Dutta

**Affiliations:** ^1^​ Department of Plant Pathology, University of Georgia, Tifton GA 31793, USA; ^2^​ Department of Plant Pathology, University of Georgia, Athens GA 30602, USA

**Keywords:** average nucleotide identity, novel species, pepper, *Pseudomonas capsici*

## Abstract

Three phytopathogenic bacterial strains (Pc19-1^T^, Pc19-2 and Pc19-3) were isolated from seedlings displaying water-soaked, dark brown-to-black, necrotic lesions on pepper (*Capsicum annuum*) leaves in Georgia, USA. Upon isolation on King’s medium B, light cream-coloured colonies were observed and a diffusible fluorescent pigment was visible under ultraviolet light. Analysis of their 16S rRNA gene sequences showed that they belonged to the genus *Pseudomonas *, with the highest similarity to *Pseudomonas cichorii* ATCC 10857^T^ (99.7 %). The fatty acid analysis revealed that the majority of the fatty acids were summed feature 3 (C_16  :  1_ ω7*c*/C_16  :  1_ ω6*c*), C_16  :  0_ and summed feature 8 (C_18  :  1_ ω7*c*/C_18  :  1_ ω6*c*). Phylogenomic analyses based on whole genome sequences demonstrated that the pepper strains belonged to the *Pseudomonas syringae* complex with *P. cichorii* as their closest neighbour, and formed a separate monophyletic clade from other species. Between the pepper strains and *P. cichorii *, the average nucleotide identity values were 91.3 %. Furthermore, the digital DNA–DNA hybridization values of the pepper strains when compared to their closest relatives, including *P. cichorii *, were 45.2 % or less. In addition, biochemical and physiological features were examined in this study and the results indicate that the pepper strains represent a novel *Pseudomonas* species. Therefore, we propose a new species *Pseudomonas capsici* sp. nov., with Pc19-1^T^ (=CFBP 8884^T^=LMG 32209^T^) as the type strain. The DNA G+C content of the strain Pc19-1^T^ is 58.4 mol%.

## Full-Text

The members of *Pseudomonas* species have been isolated from various environmental sources including soil, water, plants and animals [1]. In the past, DNA–DNA hybridization (DDH) was considered the gold standard for prokaryotic species differentiation [2]. However, DDH is laborious and a difficult approach. Sequence analysis of 16S rRNA genes and conserved housekeeping genes are often conducted for characterization of *Pseudomonas* species [3, 4], but not accepted as valid standards for bacterial species delineation. Instead, recent developments of whole-genome sequencing technologies have advanced the sequence-based taxonomy for bacteria [5].

Multiple *Pseudomonas* species and pathovars have been associated with causing diseases on a wide range of plant hosts [6]. Among the *Pseudomonas* species, *Pseudomonas syringae* has been widely studied and taxonomically well-characterized compared with other plant-pathogenic bacterial species. The multi-locus sequence analysis of housekeeping genes has been used to assign 13 phylogroups and nine genomospecies within the *P. syringae* complex [4, 6]. Due to the advances in sequencing technology, whole-genome sequences (WGS) have been used for classifying *Pseudomonas* species taxonomically [5]. Specifically, multiple pairwise comparative approaches like average nucleotide identity (ANI) and genome-to-genome distance calculations have been used for species differentiation using WGS [7]. Recently, three fluorescent *Pseudomonas* strains were isolated from symptomatic pepper foliage in Georgia, USA. Using a polyphasic taxonomic approach, we provide evidence that these strains represent a novel *Pseudomonas* species. The pepper strains Pc19-1, Pc19-2 and Pc19-3 are being proposed as *Pseudomonas capsici* sp. nov.

## Isolation and ecology

Leaf blight symptoms were observed in pepper seedlings in greenhouses in Georgia, USA in 2019. Foliar symptoms included water-soaked, dark brown-to-black, irregularly shaped lesions (Fig. S1, available in the online version of this article). Three fluorescent *Pseudomonas* strains were isolated from the symptomatic tissue of pepper leaves on King’s medium B. Pathogenicity of isolated bacterial strains was confirmed by leaf infiltration of pepper foliage (cv. Aristotle) using a syringe with bacterial suspensions containing approximately 1×10^6^ c.f.u. ml^−1^ [8]. Disease symptoms similar to the original natural infections were observed 48 h after inoculation by strains Pc19-1^T^, Pc19-2 and Pc19-3, while *P. cichorii* type strain NCPPB943^T^ did not cause necrosis on pepper leaf (Fig. S1). The standard lopat tests [9], which consist of determining levan production on 5 % sucrose medium, oxidase activity, potato soft rot ability, arginine dihydrolase assay and tobacco hypersensitivity, were conducted for preliminary characterization. The three pepper strains were positive for the oxidase test, caused potato rot (Fig. S2), and showed a hypersensitive reaction on tobacco. However, they were unable to produce levan and were negative for the arginine-dihydrolase assay. These characteristics are unique when compared to the lopat profiles of the fluorescent plant pathogenic *Pseudomonas* species reported by Lelliott *et al*. [9], but closely matched with *P. cichorii* except for the potato rot test.

## 16S rRNA gene phylogeny

**Fig. 1.**
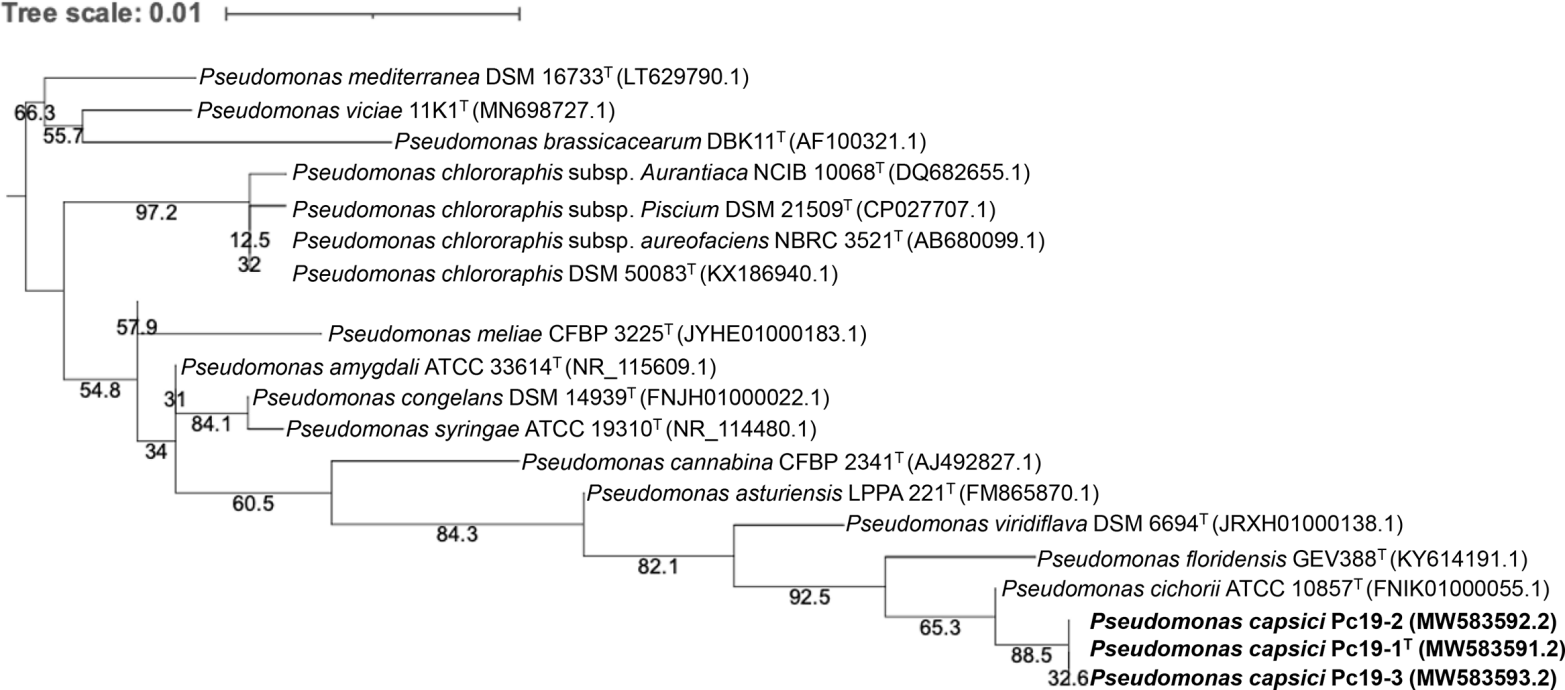
Phylogenetic relationships based on partial gene sequences of 16S rRNA between *Pseudomonas capsici* sp. nov. strains and closely related *Pseudomonas* species listed in Table 1. The 16S rRNA gene sequences (1266 nucleotides) were aligned using mafft (version 7.294b) [10]. The alignment was used to construct a phylogenetic tree using the phyml package with the maximum-likelihood method and with the best substitution model estimated by jmodelTest version 2.1.10 [12]. The clade including *Pseudomonas viciae *, *Pseudomonas brassicacearum* and *Pseudomonas mediterranea* was used for outgroup rooting. Numbers at nodes represent bootstrap values from 1000 replicates. Bar, 1 nt substitution per 100 nt. GenBank accession numbers are shown within parentheses along with the strain, with ^T^ indicating type strains.

Phylogenetic analysis was conducted using 16S rRNA gene sequences. The 16S rRNA genes of the three pepper strains was amplified using primers 27F and 1492R, and the resulting products were sequenced with primers 27F and 1492R using Sanger sequencing by Eurofins Genomics (Louisville, KY). The partial 16S rRNA gene sequences of strains Pc19-1, Pc19-2 and Pc19-3 were deposited in GenBank (accession numbers: MW583591, MW583592 and MW583593). The sequences were compared with sequences of the type strains of 17 closely related *Pseudomonas* species downloaded from NCBI. Comparative analysis revealed that the three pepper strains had identical partial 16S rRNA gene sequences and their sequence identities with *P. cichorii* type strain ATCC 10857^T^ were all 99.7 % (Table 1). The next species was *Pseudomonas ovata* F51T^T^ with 98.8 % 16S rRNA gene sequence identities. The sequences were aligned using mafft (version 7.294b) [10] within Geneious Prime 2019. A maximum-likelihood phylogenetic tree using 16S rRNA gene sequences (1266 nucleotides) was reconstructed using the phyml package [11]. The model for phylogenetic analysis was determined using the Akaike Information Criterion (AIC) statistic within jModelTest version 2.1.10 [12]. Based on the AIC, a TIM3 substitution model with invariable sites (+I) and rate variation among sites (+G) was used [13]. The robustness of the topology was estimated using 1000 bootstrap replicates. Phylogenetically, the strains were closely related to *P. cichorii* (Fig. 1).

**Table 1.** Genomic relationship between strain Pc19-1^T^ and the type strains of closely related *Pseudomonas* species as well as other *Pseudomonas capsici* sp. nov. strains The 16S rRNA gene sequences were retrieved from GenBank by similarity searches using the strain Pc19-1^T^ sequence as a query. ANIb and dDDH values were calculated using JSpeciesWS version 1.2.1 [17] and the Genome-to-Genome Distance Calculator 2.1 (formula 2) [18], respectively.

Species	16S rRNA gene sequence similarity (%)	dDDH (%)	ANIb (%)
*Pseudomonas capsici* Pc19-2	100	100	100
*Pseudomonas capsici* Pc19-3	100	100	100
* Pseudomonas cichorii* ATCC 10857^T^	99.7	45.2	91.3
* Pseudomonas viridiflava* DSM 6694^T^	97.9	25	80.6
* Pseudomonas meliae* CFBP 3225^T^	98.3	24.8	80.8
* Pseudomonas asturiensis* LMG 26898^T^	97.6	24.8	80.6
* Pseudomonas cannabina* ICMP 2823^T^	98.0	24.8	80.6
* Pseudomonas floridensis* GEV388^T^	98.4	24.8	80.5
* Pseudomonas amygdali* CFBP 3205^T^	96.9	24.7	80.5
* Pseudomonas congelans* DSM 14939^T^	98.2	24.6	80.2
* Pseudomonas syringae* KCTC 12500^T^	98.2	24.6	80.1
* Pseudomonas ovata* F51T^T^	98.8	23.3	78.4
* Pseudomonas chlororaphis* LMG 5004^T^	98.3	23.2	77.6
* Pseudomonas chlororaphis* subsp. *aureofaciens* NBRC 3521^T^	98.3	23.2	77.4
* Pseudomonas mediterranea* CFBP 5447^T^	98.2	23.1	76.8
* Pseudomonas chlororaphis* subsp. *aurantiaca* DSM 19603^T^	98.3	23	77.5
* Pseudomonas brassicacearum* LMG 21623^T^	97.6	23	76.9
* Pseudomonas chlororaphis* subsp. *piscium* DSM 21509^T^	98.2	22.9	77.4
* Pseudomonas viciae* 11K1^T^	98.0	22.8	76.8

## Genome features

The whole genomes of strains Pc19-1^T^, Pc19-2 and Pc19-3 were sequenced for taxonomic status analysis. DNA was extracted using the EZNA Bacterial DNA Kit (Omega Bio-tek). The genomic libraries were prepared using the NEBNext Ultra II DNA Library Prep Kit for Illumina and were sequenced using an Illumina Novaseq 6000 platform. The raw sequences were filtered using fastp 0.20.0 [14] and then assembled using SPAdes version 3.14 [15]. The assembled sequences were deposited at GenBank (accession numbers: JAFGZD000000000, JAFGZE000000000 and JAFGZF000000000). They were also uploaded to the Life Identification Number (LIN) platform [16]. The LINs for Pc19-1^T^, Pc19-2 and Pc19-3 are ‘50,1,0,2,0,0,0,0,0,0,0,0,0,0,0,0,0,0,0,0’, ‘50,1,0,2,0,0,0,0,0,0,0,0,0,0,0,0,0,0,0,1’ and ‘50,1,0,2,0,0,0,0,0,0,0,0,0,0,0,0,0,0,0,2’, respectively. The N50 values for strains Pc19-1^T^ (332309 bp), Pc19-2 (241 172 bp) and Pc19-3 (205290 bp) were determined. The contig numbers for the assembled sequences of Pc19-1^T^, Pc19-2 and Pc19-3 are 59, 55 and 59, respectively. The genome assembly of strain Pc19-1^T^ yielded a genome size of 5 843 696 nucleotides and its G+C content was 58.4 mol%.

The taxonomic position of the three pepper strains at the species level was determined by comparing the ANI values and digital DNA–DNA hybridization (dDDH) values between the pepper strains and 17 closely related *Pseudomonas* species. Pairwise ANI values based on blast (ANIb) were calculated between Pc19-1^T^ and phylogenetically closely related *Pseudomonas* species using jSpeciesWS version 1.2.1 [17]. The dDDH values were calculated using formula 2 of the Genome-to-Genome distance calculator 2.1 [18] using the Type Strain Genomic Server (TYGS) [19]. The calculated ANIb and dDDH values are shown in Table 1. The highest ANIb value for Pc19-1^T^ was observed with *P. cichorii* ATCC10857^T^ at 91.3 % (Table 1), which is lower than the threshold value of 95 % considered as the standard ANI value for species differentiation [20, 21]. Moreover, the dDDH value estimated for Pc19-1^T^ and the *P. cichorii* type strain was 45.2 %, which is below the 70 % threshold for prokaryotic species delineation [20, 21].

**Fig. 2.**
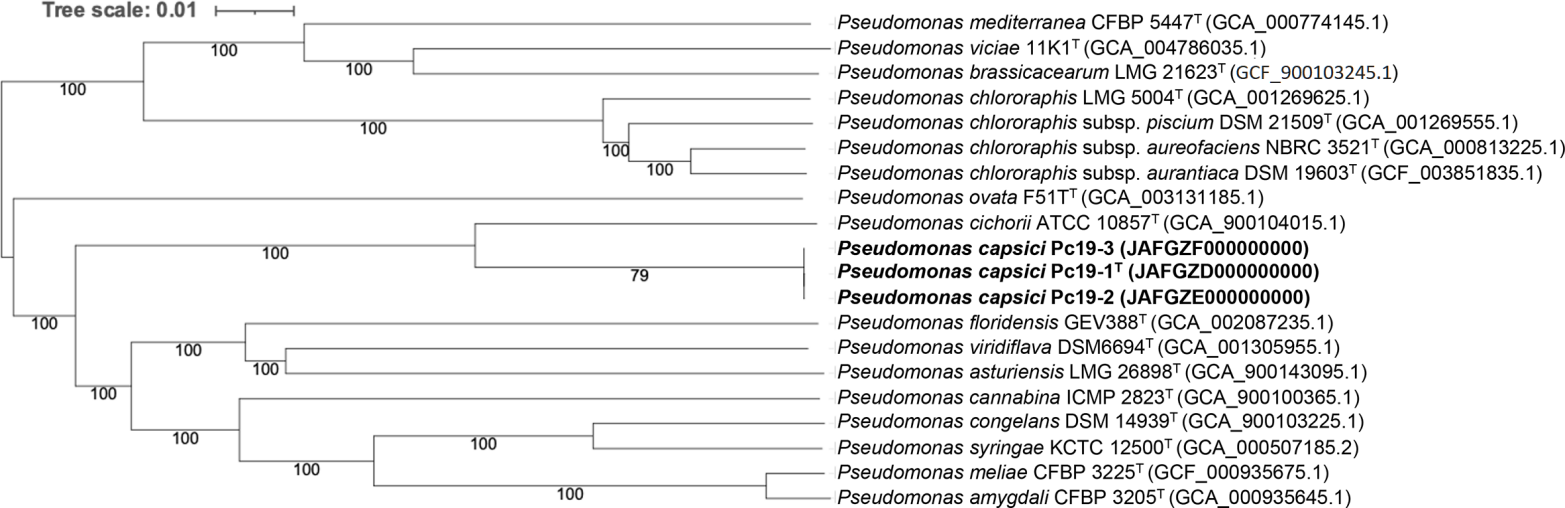
Phylogenomic relationships between *Pseudomonas capsici* sp. nov. strains and closely related *Pseudomonas* species listed in Table 1. The tree was generated with FastME 2.1.6.1 [22] from GBDP distances calculated from genome sequences on the TYGS [19]. The branch lengths are scaled in terms of GBDP distance formula d5. The numbers at nodes are genome blast distance phylogeny approach pseudo-bootstrap support values (>60 %) from 100 replications, with an average branch support of 94.9 %. The tree was rooted at the midpoint [29]. GenBank accession numbers are shown within parentheses, with T indicating type strains.

The phylogenomic analysis was carried out using the TYGS [19]. Pairwise genome comparisons were conducted using the genome blast distance phylogeny (GBDP) approach [18], and intergenomic distances were inferred under the algorithm 'trimming' and distance formula *d_5_*. The resultant intergenomic distances were used to generate a minimum-evolution tree via FastME 2.1.6.1 [22] including subtree pruning and regrafting post processing. Branch supports were inferred from 100 pseudo-bootstrap repetitions. The three pepper strains were positioned independently of the other *Pseudomonas* species and formed a monophyletic clade with a high support value of 100 % (Fig. 2). *Pseudomonas cichorii* was placed closest to the three pepper strains (Fig. 2).

To clarify the species assignment of 19 *P *. *cichorii* strains available in the NCBI genome database, we calculated the ANIb and dDDH values of these strains compared with the *P. cichorii* type strain ATCC10857^T^ and the proposed *P. capsici* type strain Pc19-1^T^ (Table 2). When comparing the strains to the *P. cichorii* type strain, four strains (REF, JBC1, MAFF301184 and ICMP6917) showed ANI values >95 % (99.0–99.9 %) and dDDH values >70 % (92.2–99.8 %), indicating they are indeed *P. cichorii *. However, when comparing to *P. capsici* type strain Pc19-1^T^, ten strains (MAFF302698, 481, Ku1409-10-1, NB15027, 482, 136, ICMP1649, 474, S-2-2-1 and 473) showed ANI values >95 % (97.2–98.5 %) and dDDH values >70 % (77.1–88.3 %), suggesting they have incorrect species-level assignations in NCBI and these ten strains deserve to be reclassified under *P. capsici*. When comparing to both *P. cichorii* and *P. capsici* type strains, the remaining five strains (Pcic4, Ku1408-5-5, MAFF302096, MAFF301764 and ICMP3353) showed ANI values <95 % and dDDH values <70 %, suggesting these five strains may be potentially new species.

**Table 2.** Genomic relationship of strains listed under *Pseudomonas cichorii* in NCBI compared to strain Pc19-1^T^ and *P. cichorii* ATCC10857^T^ ANIb and dDDH values were calculated using JSpeciesWS version 1.2.1 [17] and the Genome-to-Genome Distance Calculator 2.1 (formula 2) [18], respectively. ANIb values larger than 95 % are in bold. dDDH values larger than 70 % are in bold.

	ANIb (%)		dDDH (%)			
Strain	*P. capsici* 19-1^T^	* P. cichorii* ATCC10857^T^	*P. capsici* 19-1^T^	* P. cichorii* ATCC10857^T^	Species status	Reference
MAFF302698	**98.5**	91.3	**88.3**	45.2	*P. capsici*	[30]
481	**98.5**	91.3	**87.9**	45	*P. capsici*	N/A
Ku1409-10-1	**98.4**	91.3	**88.1**	45.2	*P. capsici*	[30]
NB15027	**98.4**	91.3	**87.9**	45.2	*P. capsici*	[30]
482	**98.4**	91.3	**87.8**	45.1	*P. capsici*	N/A
136	**98.4**	91.3	**87.8**	45.3	*P. capsici*	N/A
ICMP1649	**98.4**	91.3	**88.4**	45.3	*P. capsici*	N/A
474	**98.4**	91.3	**87.8**	45.3	*P. capsici*	N/A
S-2-2-1	**98.4**	91.3	**87.7**	45.2	*P. capsici*	[30]
473	**97.2**	91.2	**77.1**	45.3	*P. capsici*	N/A
Pcic4	92.1	94.0	48.4	57.6	Potential new species	N/A
Ku1408-5-5	92.1	94.5	48.2	59.1	Potential new species	[30]
REF	91.4	**99.9**	45.3	**99.8**	* P. cichorii *	N/A
JBC1	91.4	**99.9**	45.2	**99.8**	* P. cichorii *	[31]
MAFF301184	91.4	**99.0**	45.1	**92.2**	* P. cichorii *	[30]
ICMP6917	91.4	**99.3**	45.1	**94.4**	* P. cichorii *	N/A
MAFF302096	89.0	91.4	38.1	45.2	Potential new species	[30]
MAFF301764	88.9	91.0	37.8	43.9	Potential new species	[30]
ICMP3353	86.8	86.6	32.7	32.6	Potential new species	N/A

## Physiology and chemotaxonomy

Pc19-1^T^ grown on Luria-Bertani (LB) agar plate was cultured in LB medium at 28 °C overnight. Cell size, morphology and flagellar insertion were observed using transmission electron microscopy jeol JEM1011 at Georgia Electron Microscopy (https://gem.uga.edu/) at the University of Georgia. The bacterial cells of Pc19-1^T^ were determined to be rod-shaped with multiple polar flagella (Fig. S3). The mean cell size (±standard error) of Pc19-1^T^ was 2.4±0.2×0.7±0.1 µm (*n*=7).

The phenotypic characteristics of the three pepper strains were characterized. Carbon source utilization and tests for sensitivity to different chemicals were determined using the Biolog GEN III MicroPlate system. Bacterial cells from nutrient agar plates were suspended in Biolog inoculation fluid at 95 % transmission (optical density=0.022), and 100 µl of the suspension were added to each well of the GENIII MicroPlate and incubated at 33 °C for 22 h according to the manufacturer’s protocol. The results were recorded manually. The Biolog GENIII assay differentiated pepper strains from other closely related type strains of *Pseudomonas* species based on differences in various carbon source utilization (Table 3). The Biolog assay also showed growth at pH 5 and pH 6, and growth at 1 and 4% NaCl, but not at 8 % NaCl. API 20 NE strips (bioMérieux) were performed according to the instructions of the manufacturer. In API 20 NE assays, the pepper strains assimilated glucose, arabinose, mannose, mannitol, gluconate, caprate, malate and citrate, and were positive for nitrate reduction to nitrite, urease and β-glucosidase. The pepper strains were negative for indole production, arginine dihydrolase, gelatin hydrolysis, β-galactosidase, assimilation of *N*-acetyl-glucosamine, maltose, adipate and phenyl-acetate. Bacterial growth of the three pepper strains was observed at 4, 20, 28, 30 and 37 °C in LB medium, but not at 42 °C for a 48 h incubation period.

**Table 3.** Selected phenotypic characteristics and substrate-utilization profiles of strain Pc19-1^T^ and representative strains of closely related *Pseudomonas* species Strains: 1, Pc19-1^T^; 2, *P. cichorii* ATCC 10857^T^; 3, *P. floridensis* GEV388^T^; 4, *P. viridiflava* ICMP 2848^T^; 5, *P. asturiensis* LMG 26898^T^; 6, *P. syringae* LMG 1247^T^. All strains were negative for the production of arginine dihydrolase, and induced a hypersensitive reaction on tobacco. Data of columns 2–6 taken from Timilsina *et al.* [32]. +, Positive reaction; –, negative reaction; w, weakly positive reaction.

Characteristic	1*	2	3	4	5	6
Levan formation from sucrose	–	–	–	–	–	+
Oxidase	+	+	–	–	–	–
Potato rot	+	–	+	+	–	–
Assimilation of (Biolog GEN III):						
d-Sorbitol	–	–	+	+	–	+
l-Galactonic acid	–	–	+	–	+	w
d-Fucose	+	–	w	+	+	+
Sucrose	–	–	–	–	–	+
Troleandomycin	+	–	+	–	w	w

*Pseudomonas capsici strains Pc19-2 and Pc19-3 gave identical reactions for these tests as Pc19-1^T^.

The three pepper strains were sent to the Microbial Identification System (midi, Newark, DE, USA) laboratory to determine the cellular fatty acid composition. The cellular fatty acid content was analysed by gas chromatography (Agilent 6890 N unit) with the midi Microbial Identification System using the RTSBA6 version 6.2 library and the midi Sherlock software version 6.3 [23]. The major fatty acids were summed feature 3 (C_16  :  1_ ω7*c*/C_16  :  1_ ω6*c*), C_16  :  0_ and summed feature 8 (C_18  :  1_ ω7*c*/C_18  :  1_ ω6*c*) (Table 4). The fatty acid profiles of the pepper strains were closely related to those of *P. cichorii *.

**Table 4.** Cellular fatty acid content (%) of *Pseudomonas capsici* sp. nov. strains and related *Pseudomonas* species Strains: 1, Pc19-1^T^; 2, Pc19-2; 3, Pc19-3; 4, *Pseudomonas cichorii* DSM 50259^T^; 5, *Pseudomonas floridensis* GEV388^T^.

Fatty acid	1	2	3	4*	5†
C_10 : 0_ 3-OH	3.5	3.6	3.6	2.7	3.6
C_12 : 0_	4.9	4.8	4.9	4.1	4.7
C_12 : 0_ 2-OH	2.7	2.7	2.6	2.7	2.8
C_12 : 0_ 3-OH	4.0	4.1	4.1	3.8	4.2
C_16 : 0_	24.6	24.4	24.3	23.9	28.1
C_18 : 0_	1.0	1.0	1.0	1.1	0.6
11-methyl C_18:1_ ω7*c*	1.1	1.1	1.2	tr‡	0.8
Summed feature 3§	36.2	35.8	36.1	36.9	37.0
Summed feature 8||	22.1	21.9	21.6	23.3	16.5

*Data taken from Burr *et al.* [33].

†Data taken from Timilsina *et al.* [32].

‡tr, trace (<1 % of total).

§Summed feature 3 comprises C_16:1_ ω7*c* and/or C_16:1_ ω6*c*.

||Summed feature 8 comprises C_18:1_ ω7*c* and/or C_18:1_ ω6*c*.

In addition, analyses of polar lipids and respiratory quinones were carried out by the Identification Service of the DSMZ (Braunschweig, Germany) based on previously described methods [24–27]. The major polar lipids found in Pc19-1^T^ were diphosphatidylglycerol, phosphatidylethanolamine and phosphatidylglycerol (Fig. S4), which is consistent with species of the genus *Pseudomonas* [28]. Minor amounts of phosphatidylcholine, aminophospholipid and one unidentified lipid were also detected (Fig. S4). The major respiratory quinone was Q9 (96.9 %), which is consistent with other *Pseudomonas* species. Additional respiratory quinones detected were Q8 (1.7 %) and Q10 (1.4 %).

Considering the results of polyphasic analyses based on phenotypic characteristics, metabolic reactions, fatty acid composition, phylogenetic studies and whole-genome sequence comparisons, the three strains isolated from pepper in Georgia represent a novel species, and the name *Pseudomonas capsici* sp. nov. is proposed.

## Description of *Pseudomonas capsici* sp. nov.

*Pseudomonas capsici* (cap′si.ci. N.L. neut. gen n. *capsici*, referring to *Capsicum*, the genus name of pepper).

The colonies are light cream, opaque, round and 1.0–2.0 mm diameter after incubation at 28 °C for 24 h on nutrient agar medium. The cells are Gram-negative, aerobic, motile with multiple polar flagella and rod-shaped (2.4 µm long and 0.7 µm wide). Strains are levan-negative, oxidase-positive, positive for potato rot activity and arginine-dihydrolase-negative, and induce hypersensitive reactions on tobacco. The cells are fluorescent on King’s B medium under ultraviolet light. Cell growth occurs at 4–37 °C, with optimum growth observed between 28 and 30 °C. The bacterium grows at pH 5–6 and with 1–4% NaCl. The fatty acids comprise summed feature 3 (C_16:1_ ω7*c* and/or C_16:1_ ω6*c*), C_16: 0_, summed feature 8 (C_18:1_ ω7*c* and/or C_18:1_ ω6*c*), C_12 : 0_, C_12 : 0_ 3-OH, C_10:0_ 3-OH, C_12:0_ 2-OH, 11-methyl C_18:1_ ω7*c* and C_18 : 0_. The bacterium has diphosphatidylglycerol, phosphatidylethanolamine and phosphatidylglycerol as major polar lipids. Biolog GEN III MicroPlate assays show that Pc19-1^T^ can utilize α-d-glucose, d-mannose, d-mannitol, methyl pyruvate, γ-amino-butyric acid, d-fructose, d-arabitol, l-alanine, d-galactose, myo-inositol, d-gluconic acid, l-lactic acid, glycerol, l-aspartic acid, citric acid, d-fucose, l-glutamic acid, glucuronamide, α-keto-glutaric acid, l-fucose, d-fructose-6-PO_4_, mucic acid, d-malic acid, propionic acid, d-aspartic acid, l-pyroglutamic acid, quinic acid, l-malic acid, acetic acid, inosine, l-serine and d-saccharic acid. Strain Pc19-1^T^ is able to grow in the presence of 1 % sodium lactate, troleandomycin, lincomycin, vancomycin, nalidixic acid, aztreonam, fusidic acid, rifamycin SV, guanidine HC, tetrazolium violet, lithium chloride, d-serine, niaproof 4, tetrazolium blue, potassium tellurite and sodium bromate. Using API 20 NE assays, the bacterium assimilates glucose, arabinose, mannose, mannitol, gluconate, caprate, malate and citrate, and is positive for nitrate reduction to nitrite, urease and β-glucosidase, but negative for indole production, arginine dihydrolase, gelatin hydrolysis, β-galactosidase, assimilation of *N*-acetyl-glucosamine, maltose, adipate and phenyl-acetate. The type strain, Pc19-1^T^ (=LMG 32209^T^=CFBP 8884^T^), was isolated from symptomatic pepper foliage in Georgia, USA in 2019. The genomic DNA G+C content of the type strain is 58.4 mol%.

## Supplementary Data

Supplementary material 1
