# Supplementary material for: Pseudomonas capsici sp. nov., a plant-pathogenic bacterium isolated from pepper leaf in Georgia, USA
Source: Int J Syst Evol Microbiol. 2021 Aug 23;71(8):004971. doi: 10.1099/ijsem.0.004971 (PMC8513616; doi:10.1099/ijsem.0.004971)
Supplement: Supplementary material 1 [file ijsem-71-4971-s001.pdf]

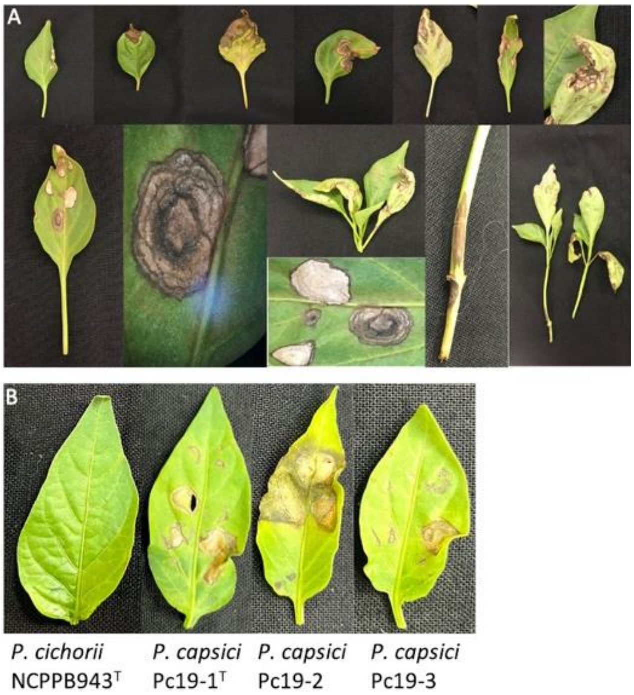


**Supplementary fig S1**. Symptoms on pepper seedlings caused by *Pseudomonas capsici* sp. nov. A) Symptoms observed in natural infections. B) Symptoms produced after artificial inoculation using *Pseudomonas cichorii* NCPPB943 $^{T}$, *Pseudomonas capsici* sp. nov. Pc19-1$^{T}$, Pc19-2, and Pc19-3 3 with bacterial suspension containing approximately 1 x $10^{6}$ CFU/ml. Pictures were taken 2 days after inoculation.

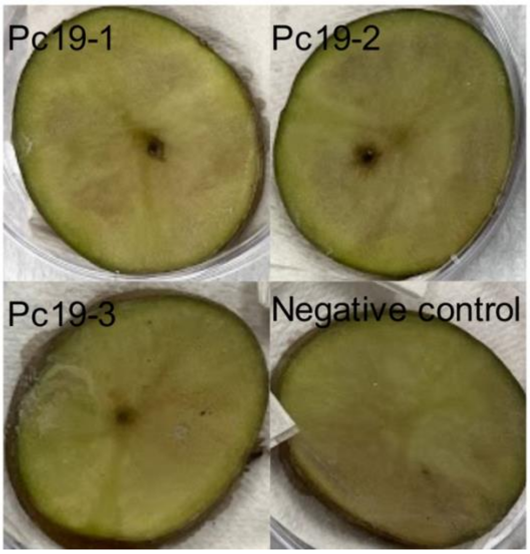


**Supplementary fig S2**. *Pseudomonas capsici* sp. nov. strains Pc19-$1^T$, Pc19-2, and Pc19-3 caused potato rot. Potato slices were stab-inoculated at the center using a sterile pipet tip touching a single colony from nutrient agar plates of strains Pc19-$1^T$, Pc19-2, and Pc19-3 and photographed 1 day after inoculation. Potato slices stabbed using a sterile pipet tip were used as a negative control.

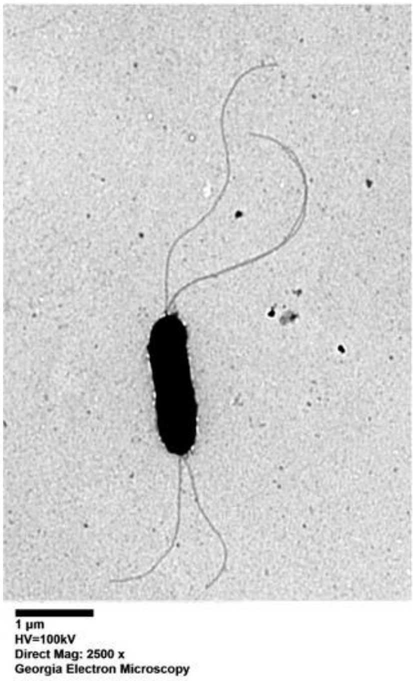


**Supplementary fig S3**. Electron microscopy image of *Pseudomonas capsici* sp. nov. strain Pc19-1$^{T}$, showing rod-shaped cells with multiple polar flagella. Bar, 1 µm.

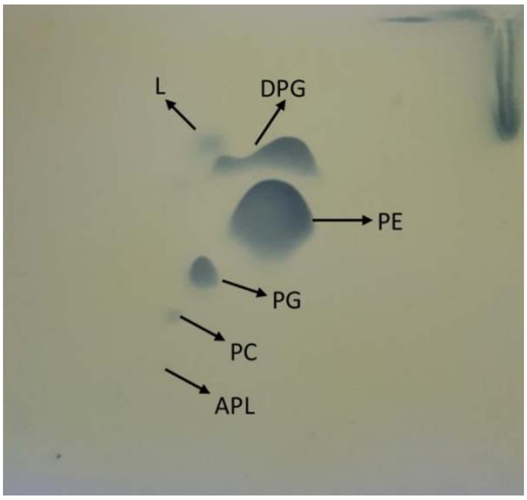


**Supplementary fig S4**. Two-dimensional thin-layer chromatography of polar lipids of *Pseudomonas capsici* sp. nov. strains Pc19-1$^T$. Abbreviations: DPG, diphosphatidylglycerol; PE, phosphatidylethanolamine; PG, phosphatidylglycerol; PC, phosphatidylcholine; APL, aminophospholipid; L, lipid.
